# Hemodynamic effects of withholding vs. continuing angiotensin II receptor blockers on the day of prone positioning spinal surgery in elderly patients

**DOI:** 10.3389/fmed.2024.1352918

**Published:** 2024-05-03

**Authors:** Ruimei Yuan, Min Xu, Chunhai Hu, Huailing Ma, Fanjun Meng, Jie Ren, Jing Wen

**Affiliations:** ^1^Department of Anesthesiology, Jinan Central Hospital, Central Hospital Affiliated to Shandong First Medical University, Jinan, Shandong, China; ^2^Department of Endocrinology, Jinan Central Hospital, Central Hospital Affiliated to Shandong First Medical University, Jinan, Shandong, China; ^3^Department of Urology, Jinan Central Hospital, Central Hospital Affiliated to Shandong First Medical University, Jinan, Shandong, China; ^4^Department of Anesthesiology, Shandong Provincial Third Hospital, Jinan, Shandong, China

**Keywords:** angiotensin II type 2 receptor blockers, norepinephrine, hemodynamics, general anesthesia, elderly

## Abstract

**Introduction:**

The hemodynamic effects of withholding vs. continuing angiotensin II receptor blockers (ARBs) before surgery in elderly patients undergoing spinal surgery in a prone position during anesthesia induction to skin incision are still unknown.

**Methods:**

In this prospective study, 80 patients undergoing spinal surgery in a prone position with general anesthesia, aged 60–79 years, American Society of Anesthesiologists (ASA) II or III, were enrolled. Patients who had ARBs only in their preoperative medication list were randomly divided into two groups at a 1:1 ratio: In Group A, ARBs were continued on the morning of surgery, while in Group B, they were withhold. Norepinephrine was infused to maintain the blood pressure at the baseline level of ±20% during anesthesia induction in all patients. The primary outcome was the consumption of norepinephrine in each group from anesthesia induction to skin incision. The secondary outcomes include changes in invasive arterial blood pressure and heart rate, the fluid infusion volumes, the amounts of anesthetic drugs, and the total time from induction to skin incision.

**Results:**

There were no significant differences in the demographics, the fluid infusion volumes, the amounts of anesthetic drugs, the total time from induction to skin incision, and hemodynamics at different time points (*p* > 0.05), while significant differences were found in norepinephrine consumption between the two groups (*p* < 0.001). Compared with Group B, the consumption of norepinephrine increased significantly in Group A (93.3 ± 29.8 vs. 124.1 ± 38.7 μg, *p* = 0.000). In addition, the same trend was illustrated in the pumping rate of norepinephrine between Group B (0.04 ± 0.01 μg·kg^−1^·min^−1^) and Group A (0.06 ± 0.02 μg·kg^−1^·min^−1^) (*p* = 0.004).

**Conclusion:**

Our study conducted in elderly patients with hypotension undergoing prone spinal surgery demonstrated a greater pumping rate of norepinephrine during anesthesia induction in patients with ARBs continuing before surgery than those withholding, indicating that it was more difficult to maintain hemodynamic stability.

**Clinical Trial Registration**: https://www.chictr.org.cn/showproj.html?proj=141081, ChiCTR2100053583.

## Introduction

In 2021, the prevalence of elderly people with hypertension in China was reported as high as 60% (1, 2), and the number of people with hypertension was increasing year by year. Preoperative hypertension has been linked to the risk of surgical bleeding, stroke, and cardiovascular complications. Long-term use of antihypertensive drugs in patients with hypertension can be combined with autonomic nervous dysfunction of regulation of blood pressure, which leads to the decrease of perioperative tolerance to hypovolemia and postural change, and sharp fluctuation of circulation, especially during the induction period of anesthesia. A retrospective study (3) found that one-third of patients undergoing non-cardiac surgery had hypotension before the skin incision, and the frequency was four times higher than that after skin incision, and postinduction hypotension was significantly associated with the prognosis of patients (4). So there are significant challenges to maintain hemodynamic stability for elderly hypertensive patients who are undergoing general anesthesia (5).

Owing to osteoporosis, trauma, and other factors, elderly patients were prone to spinal damage. Most patients require surgery to relieve pain and avoid further progression of nerve compression symptoms. The prone positional change required for spine surgery may carry a high risk of developing hypotension by a decrease in venous return from inferior vena cava compression and increased intrathoracic pressure (6, 7). In awake patients, hypotension caused by changes in position was counteracted by the baroreceptor reflex and sympathetic activation, while in anesthetized patients, anesthetic drugs can block these compensatory mechanisms and potentially increase the incidence of hypotension associated with postural change. This retrospective study (7) confirmed that the incidence of hypotension associated with supine-to-prone positional change under anesthesia was approximately three times higher than that of awake patients. Other studies (8–10) had shown that intraoperative hypotension was significantly related to poor clinical outcomes after non-cardiac surgery, especially in patients in prone position, which might lead to serious complications, such as spinal cord ischemia and postoperative vision loss (11–13).

Arterial blood pressure during anesthesia is mainly maintained by the sympathetic nervous system, arginine vasopressin (AVP), and renin–angiotensin system–angiotensin system (RAAS). When a system is suppressed, each system can be invoked as a compensation mechanism. During anesthesia, since anesthetic drugs inhibit the sympathetic nervous system, the maintenance of blood pressure mainly depends on renin–angiotensin system (RAS) or arginine vasopressin (14). Therefore, RAS is not only involved in the long-term and short-term regulation of extracellular fluid but also increases venous reflux and maintains blood pressure stability when the circulation volume is relatively insufficient (such as intravascular volume reduction or vasodilation). ARB acts as a RAS inhibitors by blocking angiotensin II, serves as a pressor, and makes patients more prone to hypotension during anesthesia (14). However, the results of many studies contradict this conclusion. Pigott et al. (15) studied prospectively 40 patients undergoing elective primary CABG surgery and found that omitting ACE inhibitors before surgery did not have sufficient advantage to be recommended routinely. Yoon et al. (16) and Turan et al. (17) also did not demonstrate any association between ACEI use and intraoperative or postoperative arterial blood pressure despite considering several definitions of hypotension and various phases of anesthesia.

So, at present, there is still controversy in clinical whether ARBs should be withhold or continued to be taken before operation (18–25). Especially for elderly patients undergoing spinal surgery in a prone position, there are limited studies on the effects of different preoperative ARB withhold strategies on hemodynamics. We hypothesized that the magnitude of the hypotensive effect of ARBs could be quantified by the amount of intraoperative vasopressor required in patients undergoing general anesthesia with a specific arterial blood pressure management. Therefore, the object of this prospective study was to investigate the total norepinephrine consumption of patients whose systolic blood pressure (SBP) was kept within 20% of the baseline and compare the effects of withholding or continuing ARBs on hemodynamic stability during anesthesia induction, so as to provide a clinical reference for preoperative ARB administration in elderly patients undergoing spinal surgery in a prone position.

## Materials and methods

This is a prospective, single-center, single-blinded, and randomized clinical trial registered in the Chinese Clinical Trial Registry (ChiCTR2100053583). Our study obtained approval from the Ethics Committee of Jinan Central Hospital (KY-2021-015-01), and all methods were conducted in accordance with the local legislation and institutional requirements. All selected 80 patients were informed about the purpose of the trial and obtained written consent. Patients whose preoperative blood pressure at the time of admission ≤140/90 mmHg and had an ARBs only in their preoperative medication list at least 3 months were randomly scheduled by using Epicalc 2000 soft and divided into two groups as a 1:1 ratio: In Group A, ARBs were continued on the morning of surgery, while in Group B, they were withhold. Inclusion criteria were patients aged 60–79 years undergoing spinal surgery in a prone position with endotracheal intubation anesthesia, ASA physical status II or III, hypertension history, and preoperative blood pressure could be well controlled (SBP ≤ 140/90 mmHg) only by ARBs at least 3 months. Exclusion criteria include (1) patients with multiple system diseases: heart failure (ejection fraction <50%), chronic liver and kidney disease (Child-Pugh Grade ≥ B; creatinine ≥177 μmol/L), and other severe heart, lung, liver, and kidney insufficiency; (2) patients with shock, moderate and severe anemia (hemoglobin <90 g/L), or severe water and electrolyte disorders; (3) patients with severe arrhythmia; (4) patients with antihypertensive drugs other than ARBs or poorly controlled preoperative blood pressure or SBP ≥ 180 mmHg or diastolic blood pressure (DBP) ≥ 100 mmHg; (5) patients with a history of hyperthyroidism; (6) patients with cerebrovascular accident or myocardial infarction in recent 6 months; and (7) patients with the surgical method temporarily changed. Sealed envelopes were selected for concealment of the group allocation at the time of patients assessed by the assistor pre-anesthesia. The assistor who evaluated the patients did not participate in anesthesia induction. Both anesthesiologists and investigators were blinded to the randomized grouping allocation.

Anesthetic management was standardized by a study protocol. All patients fasted for 8 h before operation and drank a small amount of water 2 h before operation (<5 mL/kg). The baseline blood pressure was monitored for 5 min in the ward 1 day before the operation. After entering the operating room, a peripheral venous pathway was established in the supine position. All the patients whose Allen’s tests were negative underwent radial artery cannulation with 2% lidocaine local anesthesia to monitor invasive arterial blood pressure continuously. The compound sodium chloride injection was infused 5 mL/kg 15 min before anesthesia induction and maintained at the speed of 5 mL⋅kg^−1^⋅h^−1^. All patients were induced with etomidate 0.2 mg/kg followed by sufentanil 0.4 μg/kg and rocuronium 0.6 mg/kg and were ventilated with 60% of oxygen. Then, the endotracheal intubation was completed with an electronic video laryngoscope quickly and gently within 30 s. The combination of oxygen and sevoflurane was used before the skin incision to obtain a bispectral index (BIS) between 40 and 60. Norepinephrine (2 mg, 0.03–0.1 μg·kg^−1^.min^−1^) was continuously pumped at the beginning of anesthesia induction and adjusted during anesthesia induction as needed to maintain the patient’s SBP at the preoperative baseline level of ±20%. Vital signs such as SBP, DBP, mean arterial pressure (MAP), electrocardiogram, heart rate (HR), peripheral capillary oxygen saturation (SpO_2_), and BIS were monitored. The patient underwent a supine-to-prone positional change when the hemodynamics was relatively stable.

The primary outcome was the consumption of norepinephrine in each group during induction to skin incision. The secondary outcomes include changes in arterial blood pressure and heart rate, the drug dosage of induction, the fluid infusion, and the total time from induction to skin incision. BP, HR, SpO_2_, BIS, and other vital signs were recorded at baseline (T1), pre-induction (T2), before endotracheal intubation (T3), 1 min after endotracheal intubation (T4), 1 min before in prone position (T5), 1 min after in prone position (T6), 5 min after in prone position (T7), 10 min after in prone position (T8), 15 min after in prone position (T9), and the time of skin incision (T10). Data collection included patient demographic characteristics (sex, age, height, weight, body mass index, ASA status, smoking history, previous medical history, preoperative hemoglobin and albumin, and baseline vital signs), the usage of ARBs on the day of surgery and the total time from anesthesia induction to skin incision, the total fluid infusion, and the dosage of anesthesia induction.

Based on the previous research and pre-test (16, 26, 27), on an alpha of 0.05 and a power of 90%, 37 patients were included in each group to detect a significant difference. Considering the 10% potential loss to follow-up, 40 patients were required in each group. All statistical analyses were analyzed with IBM SPSS 26.0 statistical software. All data of normal distribution were expressed as mean ± standard deviations. Kolmogorov–Smirnov test was used to assess whether the data approximately obeyed normal distribution. For those with normal distribution and homogeneity of variance, an independent sample *t*-test was used for comparison between groups. Categorical data were compared by *x*^2^ test or Fisher’s test. A *p* value or corrected *p* value of 0.05 was statistically significant.

## Results

**Figure 1:**
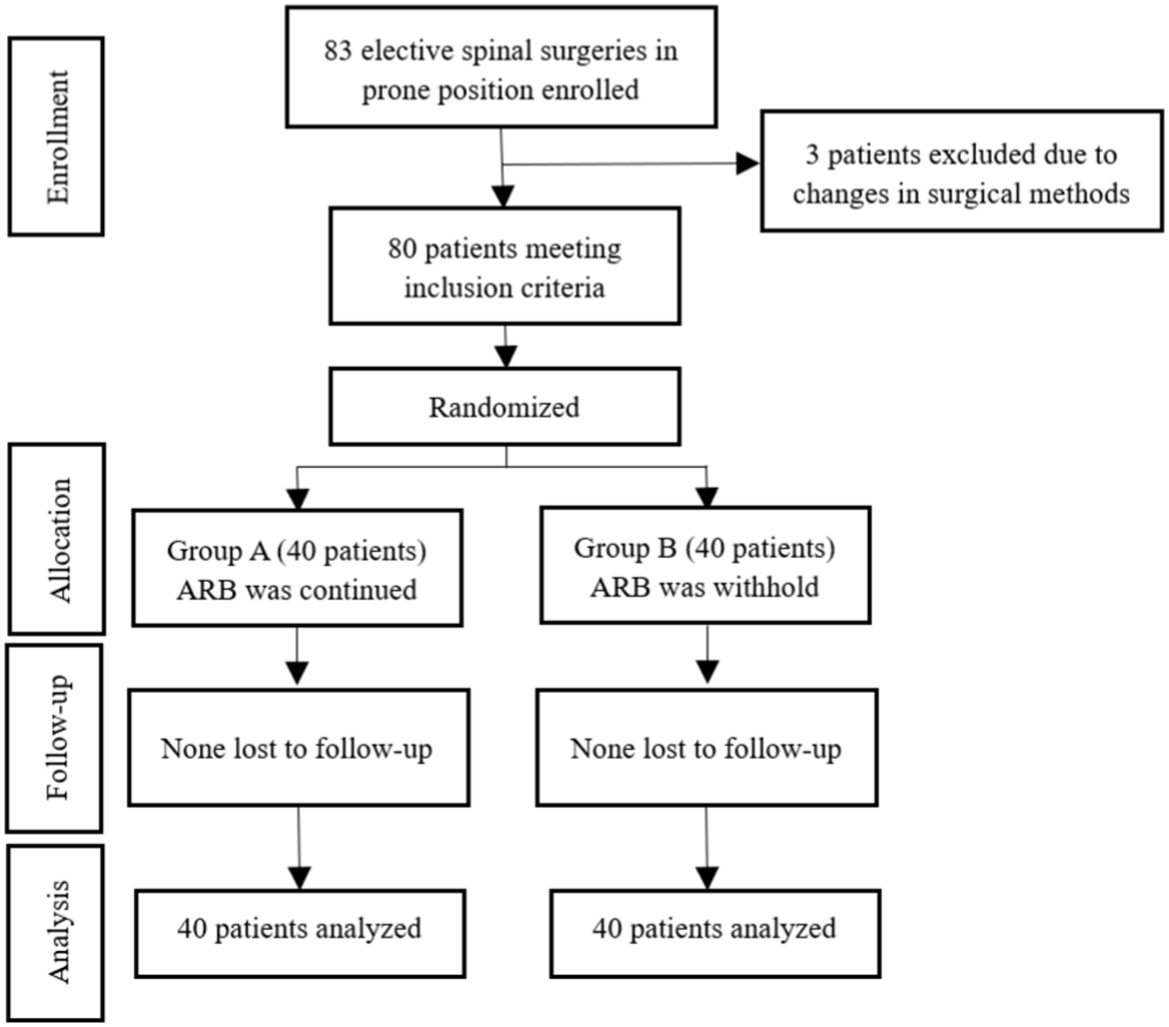
CONSORT diagram showing the distribution of patients included in our study. ARBs, Angiotensin II receptor blockers.

A total of 83 patients (from 15 April 2022 to 10 May 2023) were recruited in this clinical trial, of which three patients were excluded due to changes in surgical methods, and finally, 80 patients were included with 40 patients in Group A and 40 patients in Group B randomly (Figure 1). No significant differences were found between the two groups in demographics of gender, age, height, body weight, ASA score, history of smoking, past medical records, and preoperative hemoglobin and albumin (*p* > 0.05; Table 1).

**Table 1:** Demographic data of patients (*n* = 40 in each group).

Parameter	Group A (ARBs continued)	Group B (ARBs withhold)	*p* value
Age (years)	69.85 ± 4.29	70.45 ± 4.53	0.545
Height (cm)	164.43 ± 8.61	164.18 ± 8.76	0.898
Body weight (kg)	72.55 ± 10.38	68.20 ± 11.08	0.080
BMI (kg/m^2^)	29.83 ± 4.35	28.02 ± 4.46	0.070
Hb (g/L)	129.25 ± 16.02	127.25 ± 19.80	0.621
Albumin(g)	41.10 ± 4.67	40.14 ± 4.56	0.358
ASA score(II/III)	18/22	20/20	0.654
Sex (M/F)	19/21	18/22	0.823
Cigarette smokers (Y/N)	13/27	10/30	0.459
Diabetes mellitus (Y/N)	17/23	13/27	0.356
Coronary heart disease (Y/N)	17/23	19/21	0.653

Values are means ± SD or absolute numbers. No statistically significant differences were noted between the two groups. ARBs, Angiotensin II receptor blockers.

**Figure 2:**
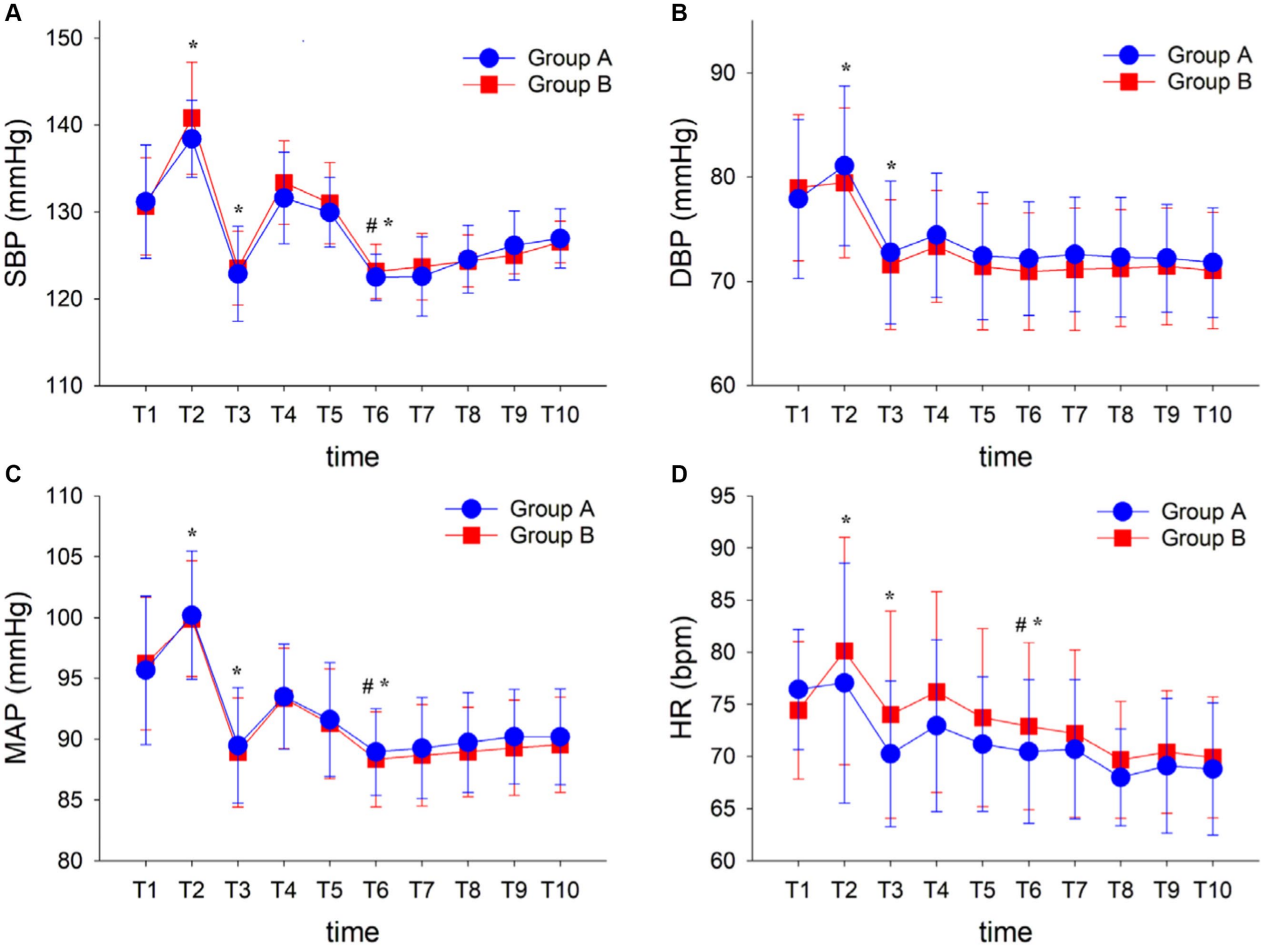
Hemodynamic variables during surgery **(A–D)**. Data are displayed as means ± SD. Time points: T1, baseline; T2, pre-induction; T3, before endotracheal intubation; T4, 1 min after endotracheal intubation; T5, 1 min before in prone position; T6, 1 min after in prone position; T7, 5 min after in prone position; T8, 10 min after in prone position; T9, 15 min after in prone position; and T10, the time of skin incision. ^*^p < 0.05 vs. T1 in each group; ^#^*p* < 0.05 vs. T5 in each group. Group A, ARBs continued; Group B, ARBs withhold; ARBs, Angiotensin II receptor blockers; SBP, Systolic blood pressure; DBP, Diastolic blood pressure; MAP, Mean artery pressure; and HR, Heart rate.

There were no significant differences in baseline vital signs (SBP, DBP, MAP, and HR) preoperative, the drug dosage of induction, and the total time from anesthesia induction to skin incision between the two groups. Despite there tends to be a higher total fluid infusion in Group A, no significant differences were found between the two groups (*p* > 0.05; Figure 2; Table 2).

**Table 2:** Characteristics of anesthesia and surgery (*n* = 40 in each group).

Parameter	Group A (ARBs continued)	Group B (ARBs withhold)	*p* value
Etomidate dosages (mg)	18.13 ± 2.70	17.13 ± 2.81	0.109
Sufentanil dosages (μg)	28.95 ± 4.27	27.30 ± 4.31	0.089
Rocuronium bromide dosages (mg)	43.63 ± 6.31	40.92 ± 6.56	0.064
Infusion volume (mL)	546.25 ± 78.14	511.63 ± 80.85	0.055
Time from induction to skin incision (min)	32.23 ± 1.59	32.20 ± 1.27	0.938

Values are means ± SD. An independent sample *t*-test was used to analyze all characteristics. ARBs, Angiotensin II receptor blockers.

In addition, no significant differences were found in hemodynamics between the two groups (*p* > 0.05; Figure 2), while significant differences were found in hemodynamics including SBP, DBP, MAP, and HR at different time points in each group. Compared with time point T1, the SBP increased at T2 and decreased relatively at T3. The SBP after the prone position (T6) was lower than that before the prone position (T5) and then slightly raised to stabilize. In addition, the same trend was noted in DBP, MAP, and HR in each time points, while all the vital signs were goaled as ±20% of the patient’s baseline values in each group (*p* < 0.05; Figure 2).

**Figure 3:**
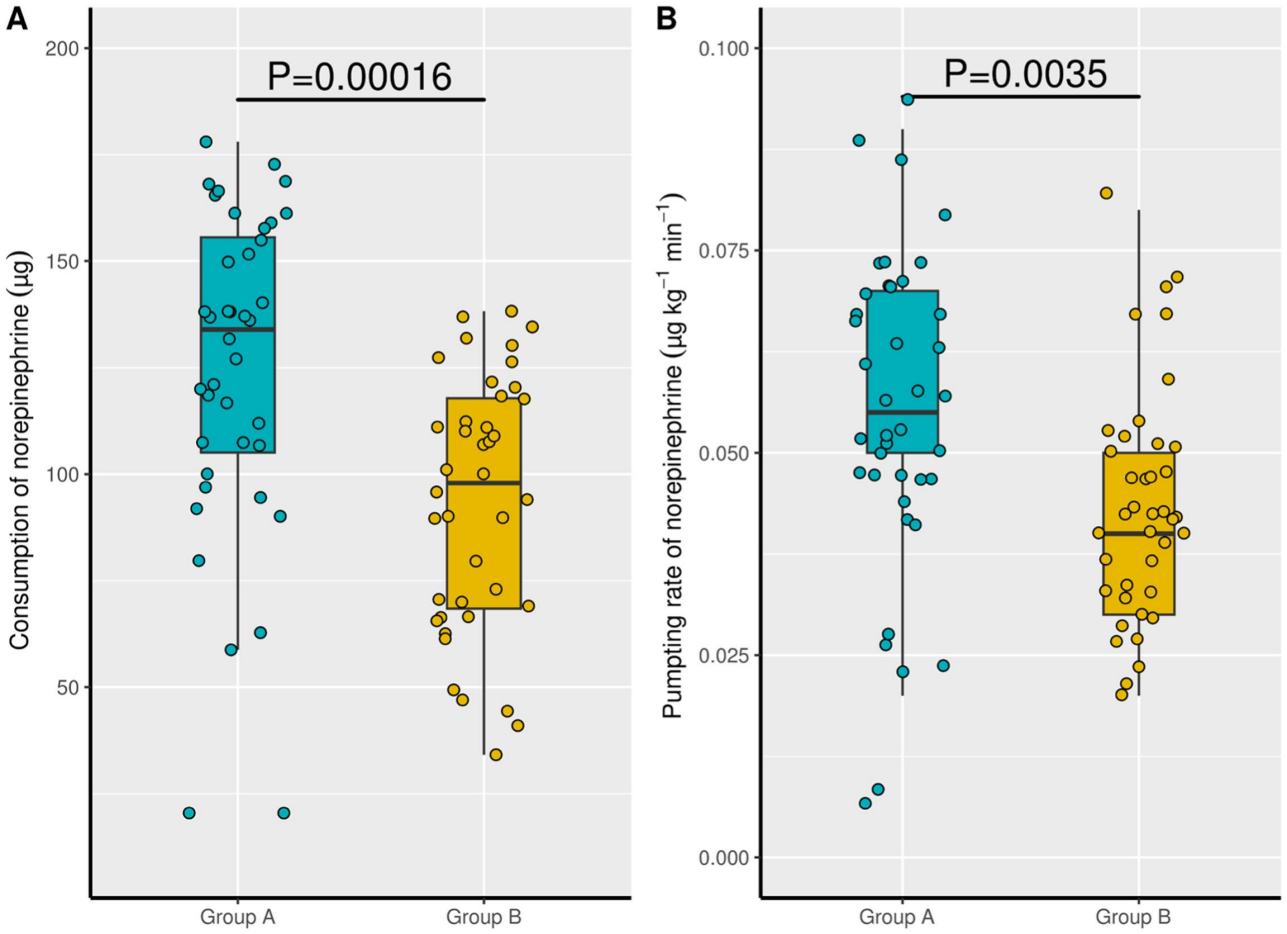
Consumption of norepinephrine and pumping rate of norepinephrine between the two groups. Group A: ARBs continued; Group B, ARBs withhold. ARBs, Angiotensin II receptor blockers.

There was a significant difference in the consumption of norepinephrine in Group A (124.1 ± 38.57 μg) and Group B (93.3 ± 29.8 μg) (*p* = 0.000). The pumping rate of norepinephrine was expressed as the consumption of norepinephrine divided by the body weight and observation time of the patients, and the same trend as consumption of norepinephrine was found between the two groups. Compared with Group B (0.04 ± 0.01 μg·kg^−1^·min^−1^), Group A (0.06 ± 0.02 μg·kg^−1^·min^−1^) had a higher pumping rate of norepinephrine (*p* = 0.004; Figure 3).

## Discussion

We designed a prospective, single-center, randomized study to explore the hemodynamic effects of withholding vs. continuing ARBs on the morning of surgery during anesthesia induction to skin incision in elderly patients undergoing spinal surgery in a prone position. In our study, it was evident that elderly hypertensive patients with continuing ARB pre-operation who underwent prone spinal surgery had a greater consumption of norepinephrine during anesthesia induction than those withholding. According to the findings, it indicated that patients with ARBs continuing pre-operation had more difficulty to maintain hemodynamic stability.

As RAS inhibitors, ARBs are crucial for short-term regulation of blood pressure maintenance and long-term regulation of intravascular fluid volume and regional circulation. The antihypertensive and beneficial cardiovascular effects of ARBs make them one of the most commonly used and effective treatments for diseases such as hypertension, congestive heart failure, coronary artery disease, and diabetic nephropathy. Each year, more than 280 million surgeries are performed globally (28), and approximately one-third are ≥45 years and are on either an angiotensin-converting enzyme inhibitor (ACEI) or ARBs before surgery (22), while perioperative use of ARBs has been reported with early postinduction hypotension in general surgery patients undergoing general anesthesia (25, 29). Further associations with these drugs include acute kidney injury in cardiovascular surgery patients (30) and increased perioperative mortality in patients undergoing coronary bypass surgery (31). Even severe postinduction hypotension (32) and cardiac arrest (33) in ACEI- and ARB-treated elderly patients in orthopedic surgery have been described. Thus, the management of patients with perioperative use of ACEIs and ARBs is especially important in the preoperative setting.

Previous studies (14, 34) reported that ARBs/ACEIs had a weak response to conventional vasoconstrictor drugs such as ephedrine and phenylephrine, especially in patients with refractory hypotension. Licker et al. (35) found that patients receiving long-term ACEI therapy could attenuate adrenergic responses without altering hemodynamic control in patients undergoing cardiac surgery. While it has been described that vasopressin and norepinephrine can effectively correct the hypotension that is refractory to conventional treatment in patients with perioperative use of RAS inhibitors (36), animal experiments also found that losartan did not decrease renal oxygenation and norepinephrine effects compared with normal rats (37). So the perioperative hypotension related to ARBs/ACEIs can be effectively treated by the application of norepinephrine. In addition, in elderly patients, due to reduced systemic blood volume, reduced organ function, and venous vascular under anesthesia, preventive application of vasoconstrictor drugs, such as norepinephrine, is recommended to maintain circulatory stability during the perioperative period and improve the patient’s postoperative outcomes (38). Therefore, our study is unique for the previous design of directly observing the incidence of hypotension in patients undergoing general anesthesia in surgery. Instead, under the premise of continuous injecting of norepinephrine, we observe the correlation between the consumption of norepinephrine and the hemodynamic effects during induction on the day of prone positioning spinal surgery in elderly patients.

For patients with long-term use of ARBs, because of the inhibition of RAAS, the incidence of intraoperative hypotension may be higher and varies from 22 to 100% according to different definitions of hypotension (18, 19). In our study, we found that elderly hypertensive patients undergoing prone spinal surgery with ARBs continuing before surgery had a greater consumption of norepinephrine during anesthesia induction than those withholding. This is not consistent with the conclusion of a previous study (39), and the reason may be related to the different inclusion criteria. In their study, a total of 26 patients who underwent bariatric surgery were enrolled, of which 14 patients received long-term ACEIs/ARBs and the remaining 12 patients had no history of hypertension. There was no significant difference in intraoperative hemodynamic changes between the two groups, and the average age of the patients was younger (average age < 47 years old) in their study. However, only elderly patients (average age > 69 years old) were included in our study, and the frequency of hypotension was more likely to occur during the perioperative period. Schulte et al. (40) illustrated that long-term ACEI treatment did not further aggravate the blood pressure decrease under TIVA during minor surgery, while in our study only the elderly patients who underwent prone spinal surgery were enrolled and they all underwent the combined intravenous and inhaled anesthesia. When elderly patients were positioned prone under anesthesia, they had a higher risk of developing hemodynamic changes due to the combined effects of prone positioning and anesthesia (41). Although the occurrence of hypotension was avoided under the continuous infusion of norepinephrine, the blood pressure of patients in the prone position still showed a downward trend compared with 1 min before the prone position, and the trend was observed obviously at 5 min after the prone position and then stabilized.

It was found that there was no statistically significant difference in blood pressure between the two groups of patients at each time. Patients in Group A had a greater demand for norepinephrine during anesthesia induction than patients in Group B. To exclude the influence of body mass index and anesthesia induction time on the observation of norepinephrine requirement (42), the pumping rate of norepinephrine in μg·kg^−1^·min^−1^ which was expressed as the consumption of norepinephrine divided by the body weight and observation time of patients was used for comparison. We still found that the pumping rate of norepinephrine was higher in Group A. The results were similar to the studies of Drenger et al. (43) and Brabant et al. (36), both of which found that continuing to take ACEIs before surgery would increase vasopressor consumption. Calloway et al. (23) demonstrated that day of surgery ACEI/ARB use was associated with a high incidence and severity of postinduction hypotension, and more ephedrine was required to maintain hemodynamic stability during surgery. Although the anesthesia method used in this study was different, their results were consistent with us. They demonstrated an increase in demand for ephedrine to maintain the stability of hemodynamics in patients who continued to take ACEIs/ARBs before surgery. Our study was unique because we presented data from elderly patients undergoing spine surgery in a prone position, performed by a small group of surgeons, with a uniform and specific hemodynamic management strategy using ABP goals as ±20% percentages of the patient’s baseline BP.

A limitation of our study is the small sample size of a specific population. A multicenter, large sample, prospective, and randomized controlled study will be needed to illustrate the hemodynamic effects of withholding vs. continuing ARBs on the day of prone positioning spinal surgery in elderly patients in the future. Also in our study, only the hemodynamic effects during the anesthesia induction period were observed. The effects of intraoperative and postoperative hemodynamics and the long-term outcomes of the patients have not been continuously observed, so further researches are needed to address this knowledge gap.

## Conclusion

Most previous studies pay their attention to the incidence or risk factors of hypotension in anesthetized patients. However, in our study, continuous infusion of norepinephrine was used during anesthesia induction to prevent patients from developing hypotension. Through comparison, the total norepinephrine consumption of patients whose BP was kept within 20% of the baseline values during anesthesia induction, we conducted that in elderly patients with hypotension undergoing prone spinal surgery demonstrated a greater pumping rate of norepinephrine during anesthesia induction in patients with ARBs continuing before surgery than those withholding, indicating that it was more difficult to maintain hemodynamic stability.

## Data availability statement

The original contributions presented in the study are included in the article/supplementary material, further inquiries can be directed to the corresponding author.

## Ethics statement

The studies involving humans were approved by the Ethics Committee of Jinan Central Hospital. The studies were conducted in accordance with the local legislation and institutional requirements. The participants provided their written informed consent to participate in this study.

## Author contributions

RY: Data curation, Investigation, Writing – original draft. MX: Formal analysis, Investigation, Writing – review & editing. CH: Data curation, Investigation, Writing – review & editing. HM: Data curation, Investigation, Writing – review & editing. FM: Methodology, Writing – review & editing. JR: Methodology, Writing – review & editing. JW: Methodology, Writing – review & editing.
